# Public Health, Public Wealth

**Published:** 1891-02

**Authors:** 


### EDITORIAL DEPARTMENT.

F. E. DANIEL, M. D., EDITOR AND PUBLISHER.

THIS JOURNAL, by unanimous vote of the members, was made the Official Organ of the Austin District Medical Society.

COLLABORATORS:

*Dr. R. M. Swearingen, Austin.*
*Prof. B. E. Hadra, M. D., Galveston.*
*Prof. Geo. Cupples, M. D., San Antonio.*
*Dr. T. C. Osborn, Cleburne, Texas.*
*Dr E. J. Doering, Chicago.*
*Dr. E. J. Beall, Fort Worth.*
*Dr Odo Betz, Germany.*
*Prof. W. B. Rogers, M. D., Memphis.*
*Dr. J. W. McLaughlin, Austin.*
*Dr. T. J. Tyner, Austin.*
*Prof. J. F. Y. Paine, M. D., Galveston.*
*Dr. R. H. L. Bibb, Mexico.*
*Dr. T. J. Bennett, Austin.*
*Dr. Bat Smith, Wharton.*
*Dr. E. Meierhof, New York.*
*L. H. Luce, M. D., West Tisbury, Mass.*

### PUBLIC HEALTH, PUBLIC WEALTH.

Away back in the centuries, when civilization was young, man recognized the importance to the community, of health. The idea crystalized into the now trite saying: "Public health is public wealth." Later, with the Romans, it became the "supreme law;" the feeble and decrepit offspring were put to death.

In 18—, Dr. Farr compiled statistics, showing the number of deaths from causes which were attributable to neglect of hygiene, upon which such reforms were at once instituted by Parliament (the celebrated and first health bill), as resulted in adding two and half years to the life of every man, woman and child in the kingdom of Great Britain; it affected the price of annuities and lowered the cost of life insurance.

That the intelligent citizens who are sent to the capital to make laws for Texas, are not totally ignorant of the importance of public health, nor totally indifferent to it, but must recognize it as the basis and foundation of public wealth, and the prosperity of the State, is evidenced by the existence of our very excellent quarantine law. This shows that they have tried to meet the emergency. But, as Dr. Eastland has pointed out in his excellent paper in this issue, they do not fully comprehend its importance, and are not acquainted with all the sources of danger which threaten us. So long as the gifted Swearingen is at the helm and our majestic and brainy Governor stands at the bridge, in command, the "ship of State" is safe from invasions of epidemic disease. But we may say, this is only one, and a minor danger;—vaccination is a sure safe-guard against small-pox, and nearly all other diseases can be controlled by proper management.

But there is a danger—greater, ten fold, than this, than these, —than all others; the danger of being murdered with entire impunity by the ignorant "Doctor." The practice of medicine should, by this Legislature, be restricted to the hands of those who can show to a legal authority that they have received such education and training as to fit them for the exercise of the grave and dangerous privilege. There is no quarantine that will keep out the Quack; fumigation and disinfectants are equally powerless, while there is no such thing as vaccinnating against him. There is no "Koch's curative lymph" that will avail against his ignorance, nor will "Pasteur's plan" prevent what is worse than rabies—the legal slaughter of the innocents at the hands of the smooth and plausible, but very incompetent self-styled "Doctor." The people. as we have often said, have no way of discriminating. How can they know the competent from the incompetent? Nothing but a severe examination at the hands of those who are honest and able, will ever separate the chaff from the wheat in the heterogeneous mass known as "the medical profession."

The Legislatures have carefully thrown around property every safeguard that words and wisdom can devise; they have provided laws to punish all other classes of people who take life, intentionally or otherwise;—even an engineer is held responsible for the lives of his cargo of living freight. A bill recently introduced assures the protection of live stock from epidemic diseases, and protects "the health of the horse, mule, ass and other stock;" and hereafter the much maligned town cow can lie down in our shrubbery beds, and chew the cud of content with a proud consciousness of being under the *ægis* of the sanitary laws of Texas, —an object of solicitude. Surely,—it must be an oversight,— something unintentional,—accident, rather than design,—that there is not on the statute books of Texas, a single provision for the protectection of the health of the *people*,—the tax-payers. and our loved and helpless families—save that relating to quarantine! Dr. Eastland has shown that, looked at from a money standpoint, a live is worth saving. Let us take a noble view of it, and enact laws to save lives—for their own sake—for humanity's sake, and the love of God. The bill to suppress quackery, now before the Senate, is backed by a petition from the people,—bearing fifteen thousand signatures,—and had opportunity offered, fifty or a hundred thousand could have been procured. Surely, the Legislature will not ignore it.

That "public health is public wealth," needs no demonstration; it has been demonstrated in all ages and in all countries. And it is a great mistake to suppose that doctors,—physicians, we mean,—thrive best where there is much sickness. The most prosperous physicians are those who live in the healthiest, and therefore the most prosperous communities. The bill to suppress quackery is a sanitary law, in as much as its object is the protection of the lives of the people from a secret and deadly danger, —one against which all other agencies are alike powerless. Every consideration of interest, economy, justice and humanity demands that a safeguard be thrown around the people to protect them against the ignorant and unscrupulous self-styled doctor, who, like Pope's fool, will "rush where angels dare not tread," and is, practically, licensed to take life, and does so with impunity. He is responsible to no tribunal,—not even to a conscience.

But this is not all that is needed: It is high time that the great State of Texas, so progressive in all else, should be applying the principles of State Medicine—that grandest of conceptions—the application of medical knowledge in the aggregate to the preservation of the public health; in a word, public sanitation. We need, and should have, by all means, a State Board of Health, in addition to our quarantine laws—a body representing the State, and a part of its government, whose duty should be a general supervision of the public health, to protect it from dangers of all kinds. We have enumerated only a few of them. There is danger lurking in every box and package of patent medicine, and in many articles of food of daily consumption.

Adulteration to cheapen, and thus increase profit, is practiced to the point not only of injuring health, but of destroying life, in many articles of prime necessity. This should be remedied. And, in order that further reforms may be instituted from time to time, it is necessary that a careful and correct record should be kept of every death and its cause. It is well known that the majority of deaths are from causes easily preventable. Registration of births, etc., should be provided for, for obvious reasons. As Dr. Cupples says, there is no record of any of our departed ever having been born, and after a generation or two passes away, there will be no evidence that they ever lived.

All happiness is built on wealth and prosperity; and health is the *sine qua non* of both.

